# Effectiveness of personalized continuous care in wound care of patients with diabetic foot ulcers

**DOI:** 10.3389/fendo.2025.1612047

**Published:** 2025-07-04

**Authors:** Yan Mi, Jing Wang

**Affiliations:** Department of Endocrinology, The Central Hospital of Enshi Tujia and Miao Autonomous Prefecture, Enshi, Hubei, China

**Keywords:** diabetic foot ulcer, personalized continuous care, psychological distress, sleep quality, wound healing

## Abstract

**Background:**

Diabetic foot ulcers (DFUs), a common complication of diabetes, are often accompanied by delayed wound healing, pain, psychological distress, and sleep disturbances.

**Objective:**

To evaluate the effectiveness of personalized continuous care (PCC) compared to routine care in improving wound healing, symptom severity, and psychological/sleep outcomes in DFU patients.

**Methods:**

A retrospective cohort study of 60 DFU patients (2021–2024) compared PCC (n=30) with routine care (n=30). Outcomes assessed included wound area reduction, granulation tissue coverage, symptom scores (ulceration, necrosis, pain), and validated psychological (SDS, SAS) and sleep (AIS) scales.

**Results:**

The PCC group showed superior wound healing (40.51% *vs*. 27.43% area reduction; 61.66% *vs*. 46.32% granulation coverage, p<0.05), lower symptom scores (ulceration: 3.18 ± 0.45 *vs*. 4.46 ± 0.6; pain: 2.01 ± 0.29 *vs*. 3.45 ± 0.58, p<0.01), and improved psychological (SDS: 32.1 ± 3.88 *vs*. 44.87 ± 4.05; SAS: 30.36 ± 3.77 *vs*. 43.25 ± 4.56, p<0.001) and sleep outcomes (AIS: 8.23 ± 0.6 *vs*. 11.33 ± 0.94, p<0.001).

**Conclusion:**

PCC enhances DFU wound healing, alleviates symptoms, and improves psychological well-being and sleep quality, supporting its integration routine clinical practice.

## 1 Introduction

Diabetes mellitus (DM) affects 537 million adults worldwide, with projections indicating an increase to 783 million by 2045 (1). In China, the prevalence of diabetes among adults has reached 12.4%, representing the largest diabetic population globally, with over 140 million people affected as of 2021 (2). Among DM patients, 15% to 25% develop diabetic foot ulcers (DFUs), which represent a primary cause of lower-limb amputation and diminished quality of life (3, 4). Chronic hyperglycemia, neuropathy, and poor blood circulation impair wound healing, while psychological stress and sleep disorders further exacerbate these outcomes (5–7). Diabetic foot disease, a complication closely associated with diabetes, is one of the leading causes of amputation (7). As such, diabetic foot ulcers represent a major public health burden that demands urgent and effective management strategies.

As a common yet severe complication, diabetic foot disease—including DFUs—imposes long-term physical and psychological burdens on patients (8–10). DFUs typically manifests as local skin rupture, ulceration, and infection in the lower extremities, potentially leading to tissue necrosis and osteomyelitis (11). In severe cases, they may necessitate amputation or even threaten life. Persistent skin damage, ulceration, and infection around the ulcer render wound healing extremely difficult. According to the latest guidelines from the International Working Group on Diabetic Foot (IWGDF 2023), effective DFU management requires a multidisciplinary and individualized approach that addresses not only wound care but also systemic and psychosocial factors (12). Management of chronic wounds represents a critical challenge in clinical care, as the healing process is influenced not only by physiological factors but also by patients’ psychological states and sleep quality. Numerous studies have demonstrated that psychological stress and depression activate the hypothalamic-pituitary-adrenal (HPA) axis, increasing cortisol secretion, which suppresses immune function and delays tissue repair (13, 14). Similarly, sleep disturbances are associated with reduced growth hormone production and elevated levels of inflammatory cytokines, such as IL-6 and TNF-α, which contribute to poor wound healing outcomes (15). Sleep disorders further exacerbate healing delays by disrupting growth hormone secretion and increasing the release of inflammatory cytokines (e.g., IL-6, TNF-α). This highlights the need for care strategies that go beyond physical treatment and address the full complexity of DFU-related complications.

Although conventional nursing models maintain wound stability through basic measures such as debridement and anti-infection therapy, they often overlook systematic interventions for patients’ psychological and sleep needs, potentially becoming a latent blind spot affecting treatment efficacy. Therefore, effective treatment and nursing care for DFUs are of paramount importance (16–18). Among various therapeutic approaches, individualized continuous nursing care is recognized as a vital component of DFU management (19). The core of individualized continuous nursing care lies in providing personalized, comprehensive, and sustained medical services to meet the nursing needs of patients at different stages and with diverse requirements. This care model emphasizes comprehensive patient assessment, formulation of targeted treatment plans based on disease characteristics and lifestyle, and continuous improvement of health status through regular follow-up and plan adjustment (20–22). Therefore, addressing emotional well-being and sleep disturbances is crucial for promoting optimal wound healing outcomes in DFU patients.

Consequently, the study focuses on adult patients with Wagner grade 3–4 diabetic foot ulcers of more than four weeks’ duration, with stable comorbidities and independent mobility. This specific population was selected to ensure homogeneity in disease severity and functional status, thereby enhancing the comparability of outcomes (23), thereby providing a more scientific basis for clinical practice (24). In this context, personalized continuous care may offer a promising solution to bridge the current gaps in DFU nursing practices.

## 2 Materials and methods

### 2.1 General information

This retrospective cohort study was conducted from January 2021 to December 2024 at the Department of Endocrinology, The Central Hospital of Enshi Tujia and Miao Autonomous Prefecture, a tertiary care facility located in a central urban area of Hubei Province, China. A total of 60 patients with diabetic foot ulcers who met the study criteria were included. The sample size (n=60) was based on available eligible cases and is consistent with similar retrospective studies, with *post hoc* analysis indicating sufficient statistical power to detect group differences and routine care were formed based on clinical care pathways in use during the study period. The group assignment was non-randomized and depended on the care model followed by the treating team. The PCC model was introduced in mid-2022 as part of a hospital-wide nursing improvement initiative; patients treated before this or who opted out received routine care. Patient data were collected through systematic review of the hospital’s electronic medical record (EMR) system. Key variables such as demographic information, wound characteristics, psychological scale scores, and treatment history were extracted using a standardized data collection form (**Table 1**). All data were anonymized before analysis. Patient identifiers were removed, and access to data was restricted to the research team to ensure confidentiality. Ethical approval was obtained from the hospital ethics committee (Approval No.: 20220618), with waived informed consent due to the retrospective nature.

**Table 1:** Comparison of general information of patients (mean ± SD or n).

Index	Personalized continuing care team	Routine care group	t/χ²	p
Average age (years)	53.28 ± 7.56	56.1 ± 6.87	0.26	0.80
Gender: (Male/Female)	17/13	16/14	-1.51	0.14
Diabetes duration (years)	8.83 ± 2.57	7.96 ± 3.32	1.14	0.26
Wound area (cm²)	8.1 ± 1.44	8.81 ± 0.93	0.03	0.13
Fasting blood glucose (mmol/L)	8.48 ± 0.86	8.51 ± 1.27	-0.12	0.91

### 2.2 Inclusion and exclusion criteria

Inclusion criteria (1): age ≥18 years (2); confirmed diagnosis of DFU with Wagner grade 3–4 ulcer lasting >4 weeks (3); stable comorbid conditions (e.g., controlled hypertension or heart disease without hospitalization in the past 3 months) (4); ability to ambulate independently without mobility aids. Exclusion criteria (1): end-stage organ failure (2); active malignancy (3); cognitive impairment or psychiatric illness interfering with participation.

### 2.3 Interventions

Patients in the personalized continuous care group receive individualized care plans tailored to their specific conditions. Specifically, these include: 1) regular wound cleaning and dressing changes, whereby personalized protocols are developed based on wound characteristics to maintain cleanliness and facilitate healing; 2) routine foot examinations, aimed at detecting and addressing lesions or abnormalities promptly to prevent complications; 3) nutritional support, involving customized dietary plans to ensure adequate nutrient intake and promote wound repair; 4) rehabilitation training, which designs exercise and physical therapy regimens to restore foot function and mobility; 5) psychological support, providing counseling to help patients manage emotional distress such as anxiety and depression during treatment; and 6) sleep guidance, offering strategies to improve sleep quality and mitigate the negative impacts of insomnia on healing.

In contrast, routine care followed the hospital’s standard DFU treatment protocol based on national clinical guidelines.: wound cleansing and dressing changes performed according to conventional protocols to maintain wound dryness and cleanliness, along with routine medications such as antibiotics and analgesics to control infection and manage pain.

### 2.4 Observation Indicators

Wound healing was assessed using two indicators: wound area reduction and granulation tissue coverage. Wound area was calculated with the formula area = πr², where r represents the radius, based on standardized clinical measurements. Granulation tissue coverage was visually estimated by trained wound care nurses during dressing changes, using a standardized assessment protocol and wound photographs. Two experienced assessors independently verified the results for consistency. Symptom severity, including ulceration, tissue necrosis (rot), and wound pain, was rated on a 5-point Likert scale, with higher scores indicating greater severity. Assessments were performed by clinical nursing staff during routine care. Psychological status was evaluated using the validated Chinese versions of the Self-Rating Anxiety Scale (SAS) and the Self-Rating Depression Scale (SDS), each containing 20 items scored on a 4-point scale. Scores ≥53 on SAS and ≥50 on SDS indicated significant anxiety and depression, respectively. These scales were administered in Chinese at baseline and after 4 weeks by trained nurses. Sleep quality was assessed with the Chinese version of the Athens Insomnia Scale (AIS), an 8-item tool covering sleep induction, awakenings, duration, quality, and daytime functioning. Each item is rated from 0 (no issue) to 3 (severe issue); total scores <4 indicate no sleep disturbance, 4–6 suggest suspected insomnia, and >6 indicate clinical insomnia. AIS was also administered at baseline and post-intervention by trained staff.

### 2.5 Statistical methods

SPSS statistical software was used for data analysis. Descriptive statistics were expressed as mean ± standard deviation, and t-test was utilized for comparing continuous variables between two groups, and the chi-squared test was used for count data comparison. In addition to p-values, effect sizes (Cohen’s d) were calculated for key continuous outcomes to assess the clinical significance of group differences. The P value for statistically significant differences was set at <0.05.

## 3 Result

### 3.1 Wound healing status

Following nursing intervention, the personalized continuous care group demonstrated significant advantages in wound healing. At the end of treatment, the wound area in the personalized continuous care group was significantly reduced, with an average reduction of 40.51% compared to a 27.43% decrease in the routine care group (p < 0.05). In terms of new granulation tissue coverage, the personalized continuous care group achieved 61.66%, while the routine care group achieved 46.32% (p < 0.05) (**Table 2**).

**Table 2:** Wound healing status (mean ± SD; % change).

Index	Personalized continuing care team	Routine care group	t	p
Wound area (%)	40.51	27.43	12.30	p < 0.05
New granulation tissue coverage area (%)	61.66	46.32	8.40	p < 0.05

### 3.2 Symptom score comparison

Following nursing intervention, the symptom scores (ulceration, gangrene, wound pain) in the personalized continuous care group were significantly lower than those in the routine care group. The ulceration and gangrene score were 3.18 ± 0.45 in the personalized continuous care group *vs*. 4.46 ± 0.6 in the routine care group (p < 0.01); the wound pain score was 2.01 ± 0.29 *vs*. 3.45 ± 0.58 (p < 0.01). Symptom improvement (ulceration, gangrene, wound pain) in the personalized continuous care group was significantly greater than in the routine care group (**Table 3**).

**Table 3:** Comparison of symptom scores (mean ± SD).

Index	Personalized continuing care team	Routine care group	t	p
Ulceration and decay (score)	3.18 ± 0.45	4.46 ± 0.6	-10.66	p < 0.01
Wound pain (score)	2.01 ± 0.29	3.45 ± 0.58	-12.19	p < 0.01

### 3.3 Patient psychological score

Following nursing intervention, the Self-Rating Depression Scale (SDS) and Self-Rating Anxiety Scale (SAS) scores in the personalized continuous care (PCC) group were significantly lower than those in the routine care group. The SDS score was 32.1 ± 3.88 in the PCC group and 44.87 ± 4.05 in the routine care group (p < 0.001); the SAS score was 30.36 ± 3.77 in the PCC group and 43.25 ± 4.56 in the routine care group (p < 0.001) (**Table 4**).

**Table 4:** Comparison of patient psychological scores (mean ± SD).

Index	Personalized continuing care team	Routine care group	t	p
SDS score	32.1 ± 3.88	44.87 ± 4.05	-12.49	p < 0.001
SAS score	30.36 ± 3.77	43.25 ± 4.56	-11.94	p < 0.001

### 3.4 Sleep score

Following nursing intervention, the Athens Insomnia Scale (AIS) score in the personalized continuous care (PCC) group decreased significantly, with a statistically significant difference compared to baseline (pre-treatment: 11.95 ± 1.11 *vs*. post-treatment: 8.23 ± 0.6, p < 0.001). By contrast, the routine care group showed no significant difference (pre-treatment: 12.32 ± 1.69 *vs*. post-treatment: 11.33 ± 0.94, *p> 0.05) (**Table 5**).

**Table 5:** Comparison of AIS insomnia scores (mean ± SD).

Index	Personalized continuing care team	Routine care group	t	p
Insomnia score before treatment	11.95 ± 1.11	12.32 ± 1.69	-0.98	0.33
Post-treatment insomnia score	8.23 ± 0.6	11.33 ± 0.94	-15.17	p < 0.001

## 4 Discussion

Diabetic foot ulcer (DFU), one of the common and severe complications in patients with diabetes, is often accompanied by challenges such as delayed wound healing, pain, psychological distress, and sleep disorders during treatment. Low-frequency ultrasound, as an adjunctive therapy for chronic wounds, has demonstrated remarkable efficacy, particularly in improving the healing rates of DFUs (25). Additionally, local insulin injections have been shown to positively influence DFU healing by promoting granulation tissue formation (26). Through a retrospective study involving 60 patients, we found that the personalized continuous care (PCC) group exhibited significant advantages in wound healing, symptom scores, psychological well-being, and sleep quality compared with the routine care group. Specifically, wound healing in the PCC group outperformed that in the routine care group. Reductions in wound area and increases in new granulation tissue coverage—key indicators of wound healing—were notably more pronounced in the PCC group. This superiority may be attributed to the comprehensive assessment of patients’ wound conditions in PCC, which enables the formulation of targeted treatment plans. Such plans include measures like regular wound debridement, dressing changes, foot examinations, nutritional support, rehabilitation training, and other interventions, all of which collectively promote the progression of the wound healing process.

In addition, patients in the personalized continuous care (PCC) group had significantly lower symptom scores than those in the routine care group, indicating that personalized continuous care can effectively alleviate symptoms such as ulceration, gangrene, and wound pain. This may be attributed to the timely diagnosis and management of symptoms in PCC, as well as its focus on patients’ psychological and sleep status, thereby improving their overall symptom burden. In terms of psychological and sleep outcomes, the PCC group exhibited significantly lower scores on the Self-Rating Depression Scale (SDS), Self-Rating Anxiety Scale (SAS), and Athens Insomnia Scale (AIS) compared with the routine care group, suggesting that personalized continuous care can notably enhance patients’ mental health and sleep quality. This improvement might stem from the psychological support and sleep guidance provided in PCC, which help patients manage emotional distress and sleep disorders during treatment, thus improvi Overall, personalized continuous care demonstrates significant advantages in the management of diabetic foot ulcer (DFU) patients, as it promotes wound healing, alleviates symptoms, and improves mental health and sleep quality. ng their mental state and sleep quality. A separate study has also demonstrated that a combined treatment regimen outperforms standard care in promoting diabetic foot ulcer healing (27), further confirming that systematic personalized care can significantly improve wound healing and quality of life in these patients. Overall, personalized continuous care demonstrates significant advantages in the management of diabetic foot ulcer (DFU) patients, as it promotes wound healing, alleviates symptoms, and improves mental health and sleep quality. These findings align with the 2023 IWGDF guidelines, which emphasize patient-centered, integrated care strategies to improve wound healing outcomes and reduce the risk of recurrence (12). However, this study has limitations, including a small sample size and a retrospective design. Additionally, the study did not evaluate indicators such as cholesterol levels, statin treatment rates for cholesterol management, glucose-lowering therapies with GLP-1 receptor agonists or SGLT2 inhibitors, and smoking status—factors that may substantially influence patient care strategies and treatment outcomes. Therefore, future research should prioritize these key indicators to conduct a more comprehensive assessment of the efficacy and safety of personalized continuous care in DFU management. Specifically, large-sample, multi-center randomized controlled trials are necessary to further validate the roles of these factors in diabetic foot ulcer care and their impact on clinical outcomes.

This study has several limitations. Notably, key confounding factors such as HbA1c levels, comorbidities, smoking status, and medication adherence were not included in the analysis due to incomplete retrospective data. These variables may influence wound healing and psychological outcomes, and their absence limits the ability to fully control for potential bias.

## Data Availability

The original contributions presented in the study are included in the article/supplementary material. Further inquiries can be directed to the corresponding author.
